# Hairy Cell Leukemia (HCL) Presenting As Severe Acute Respiratory Distress Syndrome (ARDS) With Legionella pneumophila: Coincidence or Causation?

**DOI:** 10.7759/cureus.48317

**Published:** 2023-11-05

**Authors:** Kayln D Holloway, Fnu Amisha, Ginell Post, Harmeen Goraya

**Affiliations:** 1 Internal Medicine, University of Arkansas for Medical Sciences, Little Rock, USA; 2 Pathology, University of Arkansas for Medical Sciences, Little Rock, USA; 3 Internal Medicine - Pulmonology/Critical Care, University of Arkansas for Medical Sciences, Little Rock, USA

**Keywords:** immunocompromised patient, pneumonia, hairy cell leukemia, acute respiratory distress syndrome [ards], legionella pneumophila

## Abstract

Due to a low index of suspicion coupled with specific growth conditions and non-specific clinical manifestations, *Legionella (L.) pneumophila* is a frequently misdiagnosed cause of pneumonia in immunocompromised patients, especially those with hematological malignancies. We present a case of severe acute respiratory distress syndrome (ARDS) secondary to Legionnaire’s disease in a patient with newly diagnosed hairy cell leukemia (HCL) to highlight the importance of early recognition, diagnosis, and treatment of Legionnaire's disease to reduce morbidity and mortality.

## Introduction

Hairy cell leukemia is a rare B cell neoplasm. As per the 1992-2001 SEER (Surveillance, Epidemiology, and End Results) registry, HCL accounts for 1000 new cases annually, comprising only 2% of leukemia and 1% of lymphoid leukemia in the United States [1]. *Legionella* (*L.) pneumophila* is a known cause of community-acquired and healthcare-associated pneumonia that can present classically in two forms: Legionnaire’s disease or Pontiac fever [2]. Legionnaire’s disease is defined as severe pneumonia with multisystem disease, whereas Pontiac fever is a flu-like illness that is often self-resolving [2]. Legionnaire'shas been well-recognized as an emerging opportunistic infection in immunocompromised patients, especially in settings of hematological malignancies and bone marrow transplants, but it remains frequently under or misdiagnosed despite its high likelihood of mortality evening in tertiary care settings (1-10%) [2-4]. Due to the inherent pathophysiology, patients with HCL are more prone to infections with intracellular pathogens like legionella, mycobacterium, and certain fungal and viral pathogens.

## Case presentation

**Figure 1:**
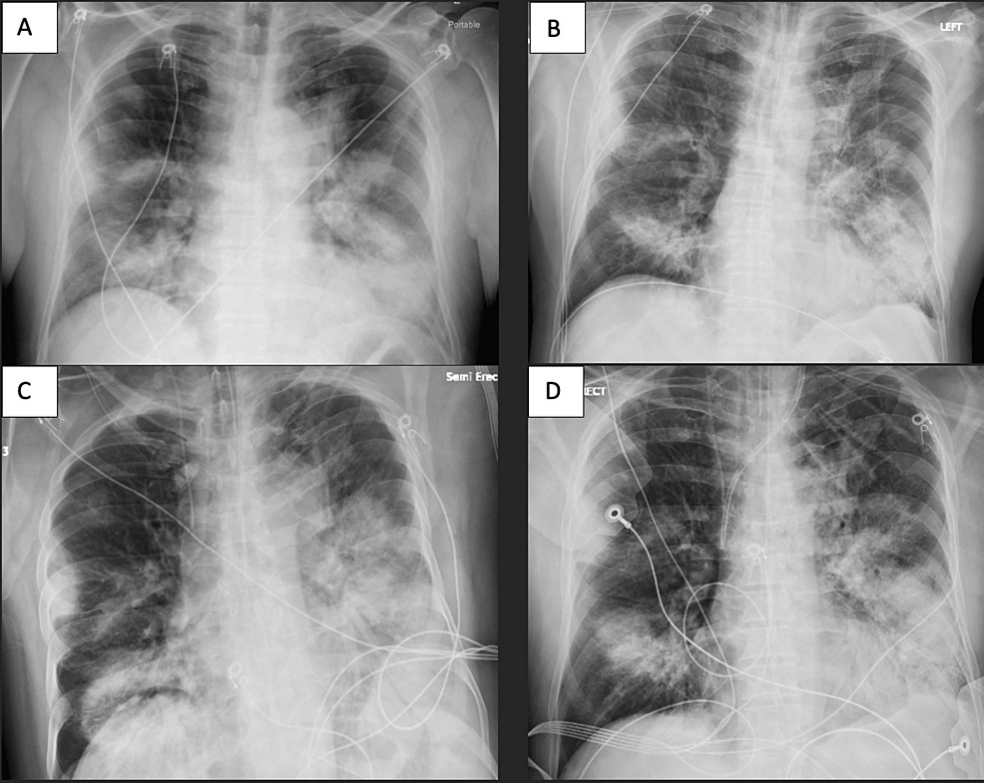
Progressive pulmonary infiltration in the setting of Legionnaire’s disease (A) Initial chest X-ray on hospital day (HD) 0 demonstrating bilateral, poorly-delineated consolidative airspace opacifications; (B) HD 1 following intubation for acute hypoxic respiratory failure – re-demonstration of consolidative bilateral multifocal airspace opacification; (C) HD 2 interval placement of a right internal jugular central venous catheter due to persistent hypotension requiring vasopressor support with X-ray demonstrating worsening infiltration versus mild pulmonary edema; (D) HD 3 demonstrating interval insertion of a left-sided Quinton catheter due to anuric renal failure and interval evolution of possible left lower pleural effusion and predominant opacification in the left mid and lower lung as well as the right lower lung.

A 59-year-old male presented to our university hospital via emergency medical services for shortness of breath with associated generalized weakness, productive cough, diarrhea, and chest tightness for six days. His other comorbid condition was alcohol use disorder. Upon arrival, he was febrile, with a temperature of 102.3 Fahrenheit, normotensive at 116/65 mmHg, tachycardic, with a heart rate of 122 beats per minute, tachypneic, with a respiratory rate of 30 breaths per minute, and hypoxic at 75% oxygen saturation (SpO_2_) on room air. Given his hypoxia and bilateral infiltrates on chest imaging, arterial blood gas analysis was obtained to evaluate for acute respiratory distress syndrome (ARDS) [3]. His Horowitz index arterial oxygen partial pressure/fractional inspired oxygen (PaO2/FiO2) ratio was 130 mmHg, indicating moderate ARDS, and he was placed on non-invasive positive pressure ventilation (NIPPV) but had quick clinical deterioration due to worsening work of breathing requiring endotracheal intubation and mechanical ventilation [5]. Initial laboratory investigations revealed macrocytic anemia, neutropenia, thrombocytopenia, hyponatremia, mild transaminitis, and a negative respiratory viral panel. Chest X-ray revealed bilateral multifocal opacifications in the lung fields (Figure 1).

**Figure 2:**
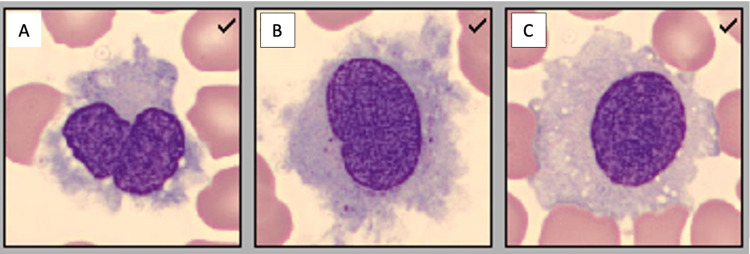
(A), (B), (C): Peripheral smear of the patient observed under CellaVision demonstrating hairy cytoplasmic projections of lymphocytes in a background of pancytopenia. The cytoplasmic projections appear thin and “hair-like” and are best visualized in panel (B). The images demonstrate a selection of the lymphocytes visible in the specimen. Images and description courtesy of Ginell R. Post, MD, PhD, in the University of Arkansas for Medical Sciences' Pathology department.

Upon admission, he was started on broad-spectrum antibiotics, vancomycin and cefepime, and when urine Legionella antigen serotype-1 testing returned positive, the antibiotic therapy was changed to levofloxacin on hospital day (HD) 2. Given bi-cytopenia, a peripheral blood smear was obtained which revealed lymphocytes with abundant cytoplasm and numerous circumferential hairy projections (Figure 2).

**Figure 3:**
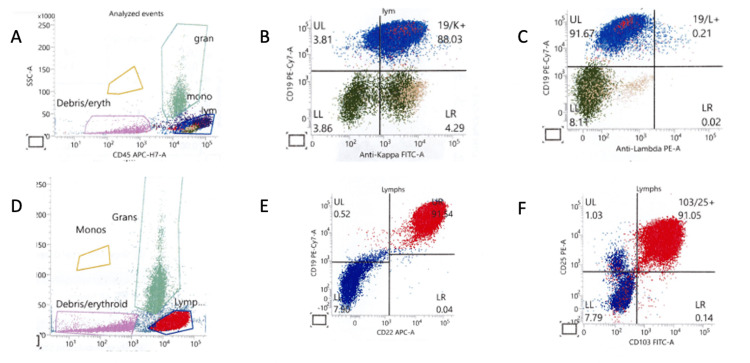
Flow cytometry of the patient’s peripheral blood with analysis demonstrating a kappa light chain restricted B cell population positive for CD19 as evidenced in panels A, B, and C with blue colored distribution. The cells also have positive CD25 and CD103, as evidenced by the red distribution in panels D, E, and F that comprised 80% of the total events. These images and description were provided courtesy of Ginell R. Post, MD, PhD, in the University of Arkansas for Medical Sciences' Pathology department.

Flow cytometric analysis of peripheral blood demonstrated a monotypic B cell population with co-expression of CD103 and CD25, consistent with hairy cell leukemia (Figure 3), consistent with HCL [6]. The clinical picture was diagnostic for Legionnaire’s disease, further confirmed with a respiratory culture, which later grew *L. pneumophila*. Given the clinical suspicion of *Legionella *infection, the microbiology lab was able to culture the sputum sample on buffered charcoal yeast extract (BYCE) media and BYCE-selective media saturated with polymixin b, vancomycin, and anisomycin incubated at 35 C for seven days. Identification was based on the selective media as well as Vitek microbial identification cards. Unfortunately, his clinical condition continued to decline and the family opted for comfort care measures on HD 5; he expired prior to the intervention for airy cell leukemia.

## Discussion

Legionella pneumophila is a facultative intracellular pathogen that replicates within aquatic protozoa in a parasitic relationship [7,8]. Infection in human hosts can occur with inhalation of the aerosolized bacterium or aspiration with water, which invade alveolar macrophages and begin replication, evading phagocytosis with a type IV secretion system that traverses the bacterial membrane and allows the injection of bacterial proteins to target cells [7,8]. Legionella outbreaks have been associated with contaminated water systems like cruise ships, hospitals, nursing homes, and hotels, and more recently with stagnant water systems amidst the COVID-19 pandemic [9]. A review of our patient's social history with his family revealed that he was a frequent fisher in the Hot Springs, Arkansas, area that is known for its natural hot springs and elevated average water temperatures.

As per a study by Htwe and Khardori in 2017, among all the hematological malignancies, HCL is most associated with *Legionella pneumophila* [10]. Mechanisms involving both B and T cells have been proposed to explain this finding. The qualitative and quantitative defect in the monocytic-macrophage system in HCL seems to play a pivotal role in this predilection. Monocytopenia in HCL is further evidenced by low serum levels of lysozyme, a low absolute number of peripheral blood monocytes, low or absent monocytes in skin-window preparations, or a sparse number of macrophages in the spleen [11]. Although not all of these features were established through testing in our patient, the absolute monocyte count was 0 K/uL on manual counts establishing the likely existence of disruption of the monocytic-macrophage system. Another pathophysiological phenomenon includes T cell dysfunction in the form of the absence of a normal CD4:CD8 ratio, absent CD 28, decreased T helper and suppressor cells, and absent antibody-dependent cellular toxicity [12,13]. Given that our patient had undiagnosed and untreated HCL with inciting environmental exposure, we believe that this predisposed him to acquire severe *L. pneumophila* pneumonia, in the form of Legionnaire’s disease, rather than this being a mere coincidence.

The etiology of community-acquired pneumonia (CAP) is usually unknown at the time of diagnosis. Therefore, the Infectious Disease Society of America (IDSA) and the American Thoracic Society (ATS) recommend that empiric treatment for severe cases should include coverage for atypical organisms like *Legionella*, *Chlamydia*, and *Mycoplasma *species with either macrolides or respiratory fluoroquinolones [14]. However, the British Thoracic Society (BTS) reserves treatment for *Legionella* for patients with moderate to severe CAP or a high index of clinical suspicion [15]. A meta-analysis done by Eliakim-Raz et al. has shown no difference in survival or clinical efficacy if atypical coverage was added to all hospitalized patients with CAP but subgroup analysis of 43 patients with CAP caused by *L. Pneumophila* revealed that the clinical failure was reduced in regimens incorporating atypical coverage without changes in total adverse events (risk ratio (RR) 0.17, 95% CI 0.05-0.63) [16]. We added levofloxacin to our patient's treatment regimen after the result for *Legionella* urine antigen was available in the setting of progressively worsening disease. The urine antigen testing for *Legionella* has been reported to be 70-95% sensitive for *L. pneumophila* serotype 1, but it is known that other serotypes are also pathologic in nature [17]. Therefore, it would have been imperative to include atypical coverage for our patient in the absence of positivity just based on the nature of severe CAP and clinical deterioration. Unfortunately, the patient expired prior to culture data becoming available, as culturing *Legionella* takes three to seven days with sensitivity <10-80% [17]. Hence, based on our clinical experience with this case, we suggest the early initiation of fluoroquinolones or macrolides on admission for atypical coverage in patients being admitted to the hospital for severe CAP and in situations with a high index of clinical suspicion. In the case of our patient, clinical suspicion was based on environmental exposure to warm aquatic areas, monocytopenia, and diagnosis of untreated HCL.

Based on the clinical course of our patient, we suggest that the prognostic markers for poor outcomes in Legionnaire's disease in HCL patients may include active and untreated HCL, advanced age, late presentation, and delay in empiric antibiotic coverage. Theoretically, using adjunctive glucocorticoids to curb the strong pro-inflammatory response, as in any other form of pneumonia, could have been possible but this has not been exclusively studied in severe *Legionella* infection. A review of the literature surrounding the adjunctive use of corticosteroids in severe CAP has been shown to improve the rate of treatment failure but has not been consistently found to improve overall mortality [18]. Another treatment modality that could have been potentially offered to our patient was the use of extracorporeal membrane oxygenation (ECMO). There have been observational studies suggesting that early referral for ECMO in patients with severe *Legionella *infection complicated by refractory respiratory failure not responding to conventional treatment was associated with better survival outcomes and patient stabilization via lung protective ventilation [19,20].

## Conclusions

Legionnaire’s disease should be considered a differential diagnosis in patients presenting with severe ARDS, especially in the background of suspicious environmental or occupational exposure or underlying evidence of hematological derangements. Early initiation of empiric coverage for atypical organisms in patients presenting with severe CAP should be done, even in the critical care setting.
